# Immunostimulatory Effects of Polysaccharides Isolated from *Makgeolli* (Traditional Korean Rice Wine)

**DOI:** 10.3390/molecules19045266

**Published:** 2014-04-23

**Authors:** Chang-Won Cho, Chun-ji Han, Young Kyoung Rhee, Young-Chul Lee, Kwang-Soon Shin, Hee-Do Hong

**Affiliations:** 1 Division of Convergence Technology, Korea Food Research Institute, Seongnam 463-746, Korea; E-Mails: cwcho@kfri.re.kr (C.-W.C.); ykrhee@kfri.re.k (Y.K.R.); yclee@kfri.re.kr (Y.-C.L.); 2 Department of Preventive Medicine, Yanbian University, Yanji 133000, China; E-Mail: hanchji@hotmail.com; 3 Department of Food Science and Biotechnology, Kyonggi University, Suwon 443-760, Korea; E-Mail: ksshin@kyonggi.ac.kr

**Keywords:** *Makgeolli*, polysaccharides, immunostimulatory activity, immunosuppression

## Abstract

*Makgeolli* is a traditional Korean rice wine, reported to have various biological functions. In this study, the immunostimulatory activity of a polysaccharide from *makgeolli* (PSM) was investigated. The polysaccharide fraction was isolated from *makgeolli* by hot water extraction, ethanol precipitation, dialysis, and lyophilization. The major constituents in PSM were neutral sugars (87.3%). PSM was composed of five different sugars, glucose, mannose, galactose, xylose, and arabinose. In normal mice, PSM treatment increased the spleen index (*p* < 0.05) as well as splenocyte proliferation (*p* < 0.05) in combination with concanavalin A or lipopolysaccharide. The immunostimulatory activities of PSM were also examined in cyclophosphamide (CY)-induced immunosuppressed mice. Mice treated with PSM exhibited increased splenocyte proliferation (*p* < 0.05), natural killer cell activity, and white blood cell counts (*p* < 0.01) compared with immunosuppressed mice. These results indicate that PSM can enhance immune function in normal mice and CY-induced immunosuppressed mice.

## 1. Introduction

*Makgeolli* is a traditional Korean rice wine that has been manufactured for more than a thousand years. *Makgeolli* is made from steamed rice with *nuruk*, which is used for saccharification, and various yeasts and lactic acid bacteria, which are used as the fermenting agent [1]. *Makgeolli* has been reported to have relatively a low alcohol content of 6%–8% and is rich in proteins and dietary fiber. *Makgeolli* also contains vitamin B complex, various organic acids, and physiologically active substances, such as inositol, acetylcholine and ten different essential amino acids [2]. *Makgeolli* has been cited for its biological activities, including its anti-cancer activity, anti-inflammatory activity, improvement of cardiovascular diseases, and suppression of preadipocyte differentiation, in addition to its nutritional value [3,4,5,6]. However, few studies have been conducted on the activity of *makgeolli* polysaccharides.

Among studies related to the physiological activities of polysaccharides obtained from the fermentation products of grains or fruits, some have explored the effect of polysaccharides from wine, soy sauce, and persimmon vinegar on immune activity. Active polysaccharides from wine and persimmon vinegar have been reported to exhibit strong anti-complementary activity [7,8]. Polysaccharides derived from traditional soy sauce have been confirmed to exhibit significant immune-promoting activity by increasing the secretion of interleukin (IL)-6 and IL-12, which are immunoregulatory cytokines of peritoneal macrophages, in addition possessing anti-complementary activity [9]. In the case of *makgeolli* polysaccharides, studies on the anti-complementary activity of various *makgeolli* and *makgeolli* *lees* polysaccharides have been reported [10,11], but studies on other immune activities are rarely found.

Recently, *in vitro* and *in vivo* experiments confirmed that polysaccharides separated from natural substances, including food and medicinal plants, can improve the immune system with relatively lower toxicity and adverse effects compared with synthetic drugs [12]. These natural polysaccharides have been known to improve immunocompetence in the body by inducing the activation of immune cells such as macrophages, lymphocytes, and natural killer (NK) cells as well as inducing the secretion of immune-related cytokines [13]. Therefore, many studies have been conducted using natural polysaccharides as immunostimulant candidates [14,15,16]. This study was performed to investigate the potential of polysaccharides from *makgeolli* (PSM) as an immunostimulator by evaluating the immunostimulatory activity of PSM in normal mice and cyclophosphamide (CY)-induced immunosuppressed mice.

## 2. Results and Discussion

### 2.1. The Chemical Properties of PSM

The chemical properties of PSM are presented in Table 1. The yield of PSM was 0.33 g/L, similar to the results (0.15–1.18 g/L) of a previous study that reported the amount of crude polysaccharides in six different types of *makgeolli* found on the Korean market [10]. PSM was composed of 87.3% neutral polysaccharides, 2.5% acidic polysaccharides, and 10.2% proteins. These results were similar to those reported by Bae *et al.*, [10] who assessed the chemical properties of polysaccharides separated from several *makgeolli* products found on the Korean market: 71.21%–95.13% neutral polysaccharides, 4.87%–6.20% acidic polysaccharides, and 0%–23.54% proteins. The analysis of sugar composition after the hydrolysis of PSM and conversion into alditol acetate derivatives revealed that among the simple sugars the glucose content was the highest at 66.4%, while mannose, galactose, xylose, and arabinose represented 24.5%, 6.6%, 1.4%, and 1.0%, respectively (Table 1).

**Table 1:** The chemical composition of PSM.

PSM		
Yield (g/L)		0.33
Chemical composition (%)	Neutral sugar	87.3 ± 0.0
	Uronic acid	2.5 ± 0.0
	Protein	10.2 ± 0.0
Composition of neutral sugar ^a^ (mole%) ^b^	Glucose	66.4 ± 0.2
	Mannose	24.5 ± 0.1
	Galactose	6.6 ± 0.0
	Xylose	1.5 ± 0.0
	Arabinose	1.0 ± 0.0

^a^ Monosaccharides were analyzed using alditol acetates; ^b^ The mole% was calculated from the total analyzed carbohydrate.

The elevated glucose content among the sugars in PSM seems attributable to the partial hydrolysis of starch in rice, the main ingredient of *makgeolli*. Pectin substances are complex polysaccharides present in plant cell walls and have been known to be made of only high molecular weight compounds (α-d-1,4-polygalacturonic acid), wherein d-galacturonic acids are connected by α-1,4 bonds. However, the pectin present in nature has been reported to exhibit much more complex structures, wherein most parts of the whole molecule are made of straight-chain homogalacturonans to which various oligo- and polysaccharides are covalently bound as rhamnogalacturonan (RG)-I and RG-II [17]. These structures have been studied in relative detail. For instance, RG-I is reported to be a polysaccharide with the main RG chain composed of galacturonic acid and rhamnose, and various side chains of arabinan, galactan, and arabinogalactan [18]. Thus, the fact that PSM contains galactose and arabinose suggests that the pectin present in rice, the main ingredient of *makgeolli*, is degraded during fermentation and that its side chains are released in *makgeolli*.

Mannose is not commonly observed in plant bodies and is notably absent from cell walls [8]. Therefore, the mannose content of PSM was assumed to be derived from the mannan of the yeast used in fermentation. Yeasts such as *Saccharomyces cerevisiae* are involved in the fermentation of *makgeolli*, and the cell wall of this yeast is largely composed of β-glucan and chitin; the outermost wall exhibits bound mannoproteins [19]. Thus, it is believed that the yeast has been degraded during fermentation process, resulting in β-glucan and chitin. These substances are precipitated due to their low solubility and the yeast cell wall-derived mannose is dissolved in *makgeolli*. PSM was eluted on HPLC equipped with a gel filtration column as a complex slowly eluted peak pattern covering a wide molecular weight range of 10–300 kDa (data not shown), suggesting the heterogeneity of PSM. Therefore, PSM is thought to be composed of heterogeneous polysaccharides from rice, the main ingredient used during fermentation, and from yeast cell walls, the agent of alcohol fermentation.

### 2.2. Effects of PSM on Immune Functions in Normal Mice

#### 2.2.1. Effects on Body and Organ Weights

PSM was administered at 100 and 200 mg/kg to normal mice, and its effects on the average body weight and the weights of the thymus and spleen are presented in Table 2. CVT-E002™, immunostimulatory polysaccharide-rich extract of the root of North American ginseng (*Panax quinquefolium*), was used as a positive control [20]. The average body weight did not significantly differ among treatment groups, suggesting that the PSM used in the experiment did not significantly affect body weight in mice. The thymus and spleen are important immune organs and their weights are recognized as important and intuitive indices for nonspecific immunity of organism [15]. Moreover, it was reported that immunostimulators could increase the weight of the thymus and spleen [21]. In this study, the spleen index significantly increased in the 200 mg/kg PSM treatment group (*p* < 0.05). The thymus index tended to increase, but the difference was not statistically significant.

**Table 2:** The effects of PSM on immune organs in normal mice.

Group	Number of animals	Initial body weight (g)	Final body weight (g)	Weight growth rate (%)	Spleen Index (SI) ^a^	Thymus Index (TI) ^b^
Control	10	22.00 ± 1.79	35.50 ± 4.38	61.36	0.65 ± 0.08	0.35 ± 0.13
CVT ^c^ 200 mg/kg	10	21.45 ± 1.90	34.66 ± 2.65	61.59	0.77 ± 0.09 *	0.42 ± 0.11
PSM 100 mg/kg	10	21.60 ± 1.90	34.88 ± 5.33	61.48	0.68 ± 0.10	0.36 ± 0.11
PSM 200 mg/kg	10	21.60 ± 1.84	34.86 ± 3.44	61.39	0.76 ± 0.13 *	0.40 ± 0.12

* *p* < 0.05, when compared with control group; ^a^ SI = Spleen weight/Body weight; ^b^ TI = Thymus weight/Body weight; ^c^ CVT: CVT-E002™ (immunostimulatory polysaccharide-rich extract of the root of North American ginseng) was used as a positive control [20].

#### 2.2.2. Effects on Lymphocytes Proliferation

**Figure 1:**
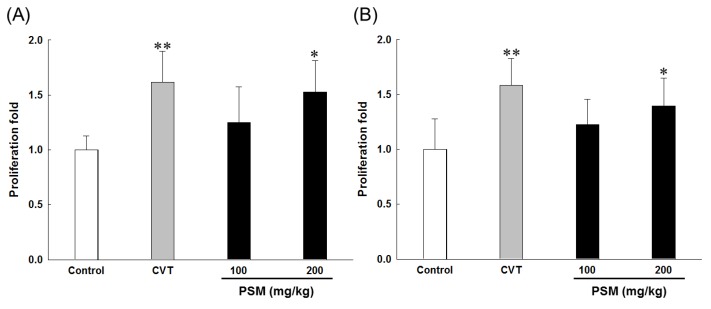
The effects of PSM on lymphocyte proliferation in normal mice. The effects of PSM on (**A**) ConA-induced T-lymphocyte or (**B**) LPS-induced B-lymphocyte proliferation. CVT: CVT-E200™ (200 mg/kg) was used as a positive control. Cell proliferation was measured by the MTT assay. The data are expressed as the mean ± SD (*n* = 10). * *p* < 0.05, ** *p* < 0.01 *vs.* vehicle-treated control group.

The proliferation of spleen cells is one of the most important steps in the activation pathway of cell-mediated or humoral immunity [14]. The immune cell proliferation in spleen cells of mice treated with PSM is presented in Figure 1. It has been well known that concanavalin A (ConA) and lipopolysaccharide (LPS) were used to stimulate T lymphocyte and B lymphocyte proliferation, respectively [22]. When the T and B lymphocyte proliferation rates were compared, PSM was confirmed to increase lymphocytes proliferation. Spleen cell proliferation ability has been widely used as a method to screen for new immunostimulators, as cell division and DNA synthesis can be stimulated by various antigens, mitogens, and cytokines [23]. This study confirmed that the administration of PSM increased the mitogen-stimulated proliferation of mouse spleen cells. Thus, PSM is considered to activate mouse lymphocytes.

### 2.3. Effects of PSM on Immune Functions in CY-induced Immunosuppressed Mice

#### 2.3.1. Effects on Lymphocytes Proliferation

**Figure 2:**
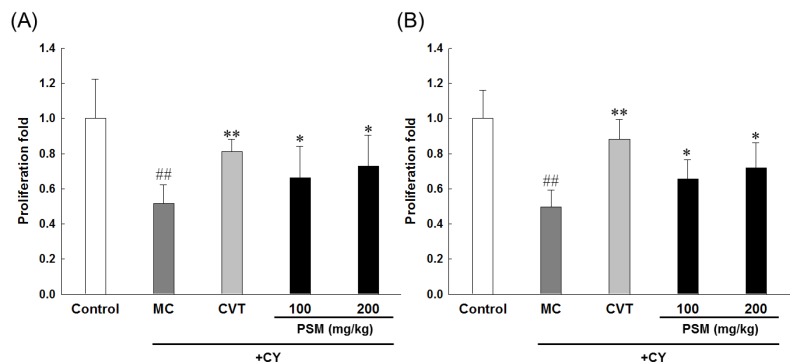
The effects of PSM on lymphocyte proliferation in CY-treated immunosuppressed mice. The effects on (**A**) ConA-induced T-lymphocyte or (**B**) LPS-induced B-lymphocyte proliferation. Model control (MC): CY-treated immunosuppressed mice. CVT: CVT-E200™ (200 mg/kg) was used as a positive control. Cell proliferation was measured by the MTT assay. The data are expressed as the mean ± SD (*n* = 10). ^#^^#^ *p* < 0.01 *vs.* vehicle-treated control group; * *p* < 0.05, ** *p* < 0.01 *vs.* CY-treated MC group.

CY is an alkylating agent used in the treatment of autoimmune diseases such as systemic lupus erythematosus, nephritis, multiple sclerosis, and rheumatoid arthritis, and CY metabolites suppress T and B lymphocytes immunity by alkylating spleen cell DNA [24]. The immune cell proliferation in spleen cells in immunosuppressed mice treated with PSM is presented in Figure 2.

The administration of 100 mg/kg CY inhibited spleen cell proliferation. This effect was consistent with prior findings that CY administration inhibited mitogen-stimulated lymphocyte proliferation [25]. In this study, T and B lymphocytes proliferation suppressed by CY administration greatly increased in the PSM treatment groups. Considering the proliferative effect of mitogen-stimulated lymphocytes in normal mice upon the oral administration of PSM (Figure 1), the restoration of CY-induced T and B lymphocytes proliferation suppression upon PSM treatment is believed to be due to the mitogen activity of PSM itself to improve lymphocyte proliferation.

#### 2.3.2. Effects on NK Cell Activity

**Figure 3:**
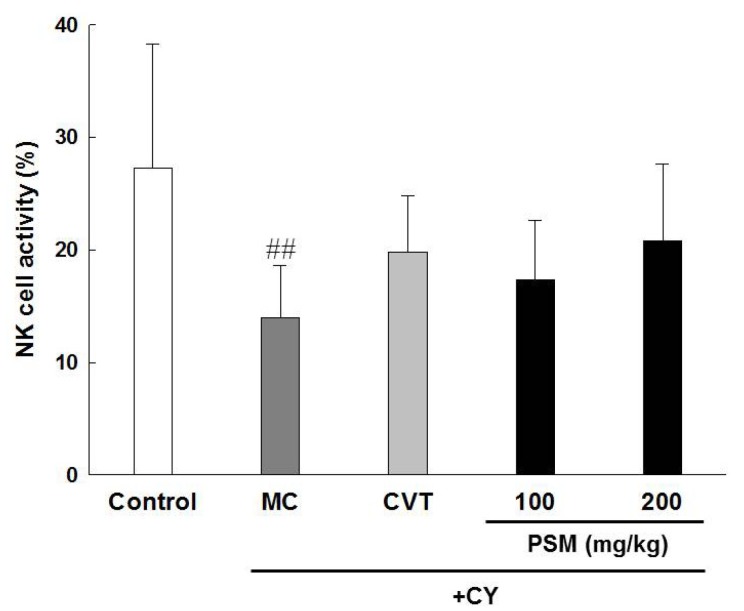
The effects of PSM on NK cell-mediated tumour cell cytotoxicity in CY-treated immunosuppressed mice. Model control (MC): CY-treated immunosuppressed mice. CVT: CVT-E200™ (200 mg/kg) was used as a positive control. The data are expressed as the mean ± SD (*n* = 10). ^#^^#^ *p* < 0.01 *vs.* vehicle-treated control group.

As a type of lymphocyte, NK cells account for 5%–15% of all peripheral blood monocyte cells and 3%–4% of spleen cells and play a key role in the innate immune system because of their ability to secrete various cytokines immediately after activation [26]. Moreover, NK cells play an important role in inducing the initial immune response against external antigens, including various viruses and bacteria, and exhibit target cell-killing activity. Therefore, the activity-stimulating effect of NK cells is considered to be reflected the state of immunity in the body [27]. The administration of CY significantly decreased the activity of NK cells compared with the control group. However, the activity of NK cells in the PSM-treated groups tended to increase, but did not differ significantly compared with the CY-treated groups (Figure 3).

#### 2.3.3. Effects on White Blood Cell (WBC) Count

**Figure 4:**
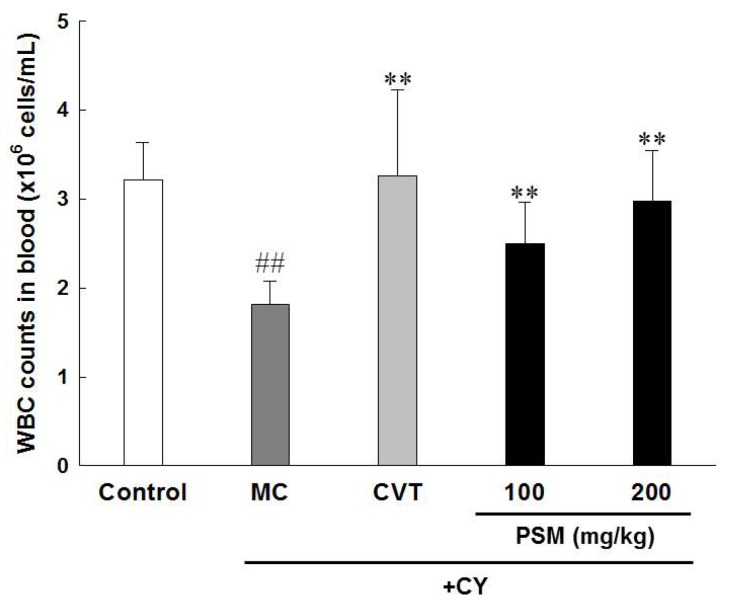
The effects of PSM on WBC counts in CY-treated immunosuppressed mice. Model control (MC): CY-treated immunosuppressed mice. CVT: CVT-E200™ (200 mg/kg) was used as a positive control. The data are expressed as the mean ± SD (*n* = 10). ^#^^#^ *p* < 0.01 *vs.* vehicle-treated control group; ** *p* < 0.01 *vs.* CY-treated MC group.

Excessive CY administration is known to decrease the numbers of leukocytes and neutrophils by immunosuppression [28]. Figure 4 presents the measurement of WBC in the blood after the oral administration of PSM for 14 days in the immunosuppressed mice model using CY. The number of WBC in the CY-treated groups significantly decreased compared with the normal group, and the PSM used in this experiment significantly increased the number of WBC, thereby suggesting that PSM can prevent CY-induced leukopenia. Because the reduction in the WBC count upon CY administration can cause severe immunodepression, various drugs have been developed to prevent leukopenia. In particular, cytokines such as granulocyte macrophage-stimulating factor and IL-11 are known to increase the number of WBC but not the overall immune responses [29]. Thus, it is very important to develop immunostimulators that can improve both the WBC count and the overall immune responses. As such, PSM has the potential to restore the immune activity inhibited by CY administration because it simultaneously increased lymphocyte proliferation, NK cell activity, and WBC count in CY-induced immunosuppressed mice.

## 3. Experimental

### 3.1. Materials

Commercial *makgeolli* samples were obtained from Seoul Takju Co. (Seoul, Korea). LPS, 3-(4,5-dimethylthiazol-2-yl)-2,5-diphenyltetrazolium bromide (MTT), ConA, dimethyl sulfoxide (DMSO), trifluoroacetic acid (TFA), and trypan blue solution (0.4%) were purchased from Sigma-Aldrich (St. Louis, MO, USA). Rosewell Park Memorial Institute (RPMI)-1640 medium was obtained from GIBCO BRL (Grand Island, NY, USA), and CVT-E002™ (sold commercially as COLD-FX^®^) was purchased from Afexa Life Sciences Inc. (Edmonton, AB, Canada). Fetal bovine serum (FBS) was obtained from Zhejiang Tianhang Biological Technology Co. (Hangzhou, China), and CY was purchased from Jiangsu Heungrui Medicine Co. (Lianyungang, China).

### 3.2. Preparation of PSM

Commercial *makgeolli* was homogenized using a food mixer (HMF-3300H, Hanil, Seoul, Korea) and then extracted at 100 °C for 30 min. Extracts were filtered through filter paper (8 µm; Whatman, Maidstone, UK) to remove insoluble particles. Polysaccharides were precipitated from extracts by the addition of four volumes of 95% ethanol. The precipitate was dissolved in distilled water and dialysed using Spectra/Por (MWCO; 6000–8000, Spectrum Laboratories Inc., Rancho Dominquez, CA, USA) for 3 days. Finally, the high molecular weight solution was lyophilized. The lyophilized powder (denoted as PSM) was stored in dark at 4 °C.

### 3.3. Characterization of PSM

Total carbohydrate, uronic acid, and protein contents were measured using phenol-sulphuric [30], carbazole-sulphuric [31] and the Bradford method [32] using glucose, galacturonic acid and bovine serum albumin as the standards, respectively. The composition of neutral sugars was determined as follows by the method partially modified from the previous method [33]. Samples were hydrolyzed with 2 mol/L TFA for 90 min at 121 °C, converted into their corresponding alditol acetates, and analyzed by gas chromatography (GC; ACME-6100, Young-Lin Co. Ltd., Anyang, Korea). The GC was equipped with an SP-2380 capillary column (0.25 mm × 30 m, 0.2 μm film thickness; Supelco, Bellefonte, PA, USA) and a flame ionization detector (FID; Young-Lin Co. Ltd.). N_2_ was used as a carrier gas at a flow rate of 1.5 mL/min. The injection temperature was 250 °C, and the detector temperature was 270 °C. The column temperature was gradually increased at 30 °C/min from 60 °C to 220 °C then at 8 °C/min from 220 °C to 250 °C. The mole percentage of each component sugar was calculated from the peak areas and response factors for FID.

### 3.4. Animals

Specific pathogen-free KM male mice (7–8 weeks) were obtained from the Experimental Animal Center of Yanbian University College of Medicine (Yanji, China). Mice were maintained under constant conditions (temperature: 22 ± 2 °C, humidity: 40%–60%, light and dark cycle: 12 h) and allowed free access to water and food.

### 3.5. Animals Experiments

Animal experiments were conducted by using both healthy and immunosuppressed mice. All animal experiments were performed according to the instructions of the Ethics Committee for Use of Experimental Animals at Yanbian University. For animal experiments with the healthy mice, the mice were randomly divided into four groups (ten mice in each group). From day 1 to 28, the four different groups of mice were orally treated the following: control group, saline; PSM groups, 100 or 200 mg/kg body weight PSM; and positive control group, 200 mg/kg body weight CVT-E002™ respectively, on a daily basis. After 24 h from the last administration, mice were weighed and sacrificed by cervical dislocation. The spleen and thymus were immediately removed and weighted. The spleen and thymus index were calculated as the spleen and thymus weight/body weight, respectively. The spleen sample was used for splenocyte proliferation.

For the experiments with the immunosuppressed mice, the mice were randomly divided into five groups (ten mice in each group). One group of mice was used as a normal control without any treatment for immunosuppression. The other four groups of mice were subjected to immunosuppression by the administration of CY (100 mg/kg/d) intraperitoneally, from day 1 to 3. Among them, one group of CY-treated mice was used as an immunosuppressed model control (MC) group. From day 4 to 17, the five different groups of mice were administered the following: control group, saline; immunosuppressed MC group, saline; PSM groups, 100 or 200 mg/kg body weight PSM; and positive control group, 200 mg/kg body weight CVT-E002™. All mice were treated daily by oral administration. After 24 h from the last administration, blood was collected from mice for WBC counts. Mice were subsequently weighed and sacrificed by cervical dislocation. The collected spleen samples were used to measure splenocyte proliferation and NK cell activity. WBC number was measured using a semi-automatic blood cell analyzer (KCTH04, Kingcare Medical Equipment Co., Ltd., Guangdong, China).

### 3.6. Splenocyte Proliferation Assay

Spleens obtained from the mice sacrificed under aseptic conditions were washed with RPMI 1640 medium and crushed to isolate spleen cells. The spleen cell mass was passed through a 200 mesh stainless steel sieve to obtain a homogeneous cell suspension. The spleen cell suspension was washed twice with RPMI 1640-FBS (containing 10% FBS) medium and with centrifugation at 1000 rpm for 5 min. The recovered spleen cells were resuspended in tris-buffered ammonium chloride solution (NH_4_Cl, pH 7.2) for 5 min to remove erythrocytes. After centrifugation, harvested spleen cells were resuspended in RPMI 1640-FBS medium, and the cell numbers were measured with a hemocytometer using trypan blue dye exclusion. Spleen cells were seeded in 96-well plates (3.0 × 10^5^ cells/well) for cell proliferation assays with ConA (5 μg/mL) or LPS (10 μg/mL). After the incubation, MTT (0.5 mg/mL) was added to each well and incubated for 4 h, and then DMSO solution was added to resolve formazan. The absorbance was measured in a microplate reader (Molecular Devices, Sunnyvale, CA, USA) at 540 nm.

### 3.7. NK Cell-Mediated Cytotoxicity Assay

NK cell-mediated cytotoxicity was measured in YAC-1 cells (NK-sensitive cell line) and primary cultured spleen cells from PSM-treated mice. Spleen cell suspensions were added to the YAC-1 cells (1 × 10^5^ cells/mL) to obtain an effector-to-target cell ratio of 50:1 in 96-well U-bottom culture plates, after which the cells were incubated at 37 °C in a humidified atmosphere containing 5% CO_2_ for 4 h. Following incubation, the culture supernatants were mixed with a lactate dehydrogenase solution (Roche, Mannheim, Germany), and the absorbance value of each well was measured at 490 nm. The NK cell cytotoxicity was calculated as a percentage as follows: cytotoxicity (%) = [(experimental release − spontaneous release)/(maximum release − spontaneous release)] × 100.

### 3.8. Statistical Analysis

All statistical analyses were performed using the Statistical Package for Social Sciences (SPSS) version 17.0 (SPSS Inc., Chicago, IL, USA). Quantitative data were expressed as the mean ± SD. All statistical comparisons were performed using a one-way ANOVA test followed by Duncan’s multiple range tests. *P*-values less than 0.05 were considered statistically significant.

## 4. Conclusions

The polysaccharide fraction PSM was separated from *makgeolli* (traditional Korean rice wine), and the immunostimulatory activity of PSM was measured in normal and immunosuppressed mice models to develop a novel bioactive polysaccharide material. PSM was mainly composed of neutral sugars, and the main components of the neutral sugars were glucose and mannose. From these results, PSM is assumed to be composed of polysaccharides from rice, the main ingredient during fermentation, and from the yeast cell wall during the process of alcohol fermentation. PSM treatment increased the weight of immune organs, the spleen and the thymus, in the normal mice model and improved the spleen lymphocyte proliferation. Likewise, PSM markedly increased the lymphocyte proliferation, NK cell activity, and WBC count in the CY-induced immunosuppressed mice model. This is the first time such PSM have shown their immunostimulatory activities in animal models. Based on the results of this experiment, PSM may exhibit potential as immunostimulators by increasing immune responses in both normal and CY-induced immunosuppression models, and further studies are needed to elucidate the mechanisms responsible for its immunostimulatory activities.
